# Effect of sevoflurane on systemic and cerebral circulation, cerebral autoregulation and CO_2_ reactivity

**DOI:** 10.1186/s12871-019-0784-9

**Published:** 2019-06-19

**Authors:** Marianna Juhász, Levente Molnár, Béla Fülesdi, Tamás Végh, Dénes Páll, Csilla Molnár

**Affiliations:** 1 Faculty of Medicine, Department of Anesthesiology and Intensive Care, University of Debrecen, Nagyerdei krt. 98, Debrecen, Hungary; 2 Faculty of Medicine, Department of Medicine, University of Debrecen, Debrecen, Hungary; 3 Hungarian Center, Outcomes Research Consortium, Cleveland, USA

**Keywords:** Sevoflurane, Cerebral blood flow, cerebral autoregulation, CO_2_-reactivity, applanation tonometry, Transcranial Doppler

## Abstract

**Background:**

Sevoflurane is one of the most frequently used inhaled anesthetics for general anesthesia. Previously it has been reported that at clinically used doses of sevoflurane, cerebral vasoreactivity is maintained. However, there are no data how sevoflurane influences systemic and cerebral circulation in parallel. The aim of our study was to assess systemic and cerebral hemodynamic changes as well as cerebral CO_2_-reactivity during sevoflurane anesthesia.

**Methods:**

Twenty nine patients undergoing general anesthesia were enrolled. Anesthesia was maintained with 1 MAC sevoflurane in 40% oxygen. Ventilatory settings (respiratory rate and tidal volume) were adjusted to reach and maintain 40, 35 and 30 mmHg EtCO_2_ for 5 min respectively. At the end of each period, transcranial Doppler and hemodynamic parameters using applanation tonometry were recorded.

**Results:**

Systemic mean arterial pressure significantly decreased during anesthetic induction and remained unchanged during the entire study period. Central aortic and peripherial pulse pressure and augmentation index as markers of arterial stiffness significantly increased during the anesthetic induction and remained stable at the time points when target CO_2_ levels were reached. Both cerebral autoregulation and cerebral CO_2_-reactivity was maintained at 1 MAC sevoflurane.

**Discussion:**

Cerebral autoregulation and CO_2_-reactivity is preserved at 1 MAC sevoflurane. Cerebrovascular effects of anesthetic compounds have to be assessed together with systemic circulatory effects.

**Trial registration:**

The study was registered at http://www.clinicaltrials.gov, identifier: NCT02054143, retrospectively registered. Date of registration: February 4, 2014.

## Background

The most important vasoregulatory mechanisms determining brain circulation are cerebral autoregulation and cerebral vasoreactivity. Cerebral autoregulation is the inherent ability of the brain circulation to maintain constant cerebral blood flow during alterations of systemic blood pressure and cerebral perfusion pressure. Cerebral vasoreactivity is responsible for enabling to change the blood flow (either globally or locally) according to the metabolic needs of the brain tissue [1]. Ideally, anesthetic agents should not have any significant impact on cerebral autoregulation and cerebral vasoreactivity during general anesthesia, which is especially important in the neurosurgical practice. There are data indicating that volatile anesthetics may alter both autoregulation and cerebral vasoreactivity to carbon-dioxide [2–4] under general anesthesia. Inhalational agents are usually characterized by dose-dependent depressive effect on cerebral autoregulation, with the exception of sevoflurane [3]. Sevoflurane, the most widely used inhalational agent does not influence cerebral vasoreactivity at MAC levels between 0.3 and 1.5 in healthy individuals [5]. There are reports suggesting that the autoregulatory plateau is shortened under sevoflurane anesthesia with MAC 1, and the upper and lower limits of autoregulation were independent of the age of the patients [5].

During induction and maintenance of general anesthesia different systemic and cerebral effects of the anesthetic agents have to be taken into account to have a global view on the anesthetic’s effect and for the interpretation of the results. Thus, in the present study we aimed to assess changes in cerebral circulation and peripheral systemic hemodynamic changes in parallel during induction and maintenance of anesthesia with sevoflurane and the effect of different hypocapnic levels.

Applanation tonometry, a new, non-invasive method has been recently reported to be accurate for non-invasive intraoperative assessment of systemic hemodynamic changes during general anesthesia [6]. In line with this, for our measurements we used parallel assessment of systemic and cerebral circulation with the combination of applanation tonometry and transcranial Doppler.

We aimed to answer the following study questions:

- What is the effect of anesthetic induction and reaching the steady state sevoflurane anesthesia on systemic hemodynamics and cerebral blood flow measurements?
- Is cerebral autoregulation maintained at normocapnic steady state 1 MAC sevoflurane anesthesia?
- What are the cerebral and systemic hemodynamic effects of induced hypocapnia at 1 MAC sevoflurane?

## Methods

### Study population

After approval from the local Ethics Committee (DEOEC RKEB/IKEB 3584–2012;), written informed consent was obtained from 35 ASA I-II patients scheduled for varicectomy and inguinal hernial repair surgery. The study was registered at http://www.clinicaltrials.gov, identifier: NCT02054143, retrospectively registered. Exclusion criteria were age < 18 years or > 60 years, severe cardiovascular disease, severe carotid artery stenosis, cerebrovascular disease, smoking, diabetes mellitus, renal disease, hyperlipidemia, left ventricular hypertrophy and severe alteration of the preoperative pulmonary function, characterized as FEV1 < 70% and FEV1/FVC ≤70% of the predicted value.

### Devices and measurements

For applanation tonometry SphygmoCor (AtCor Medical, Sydney, Australia) device was used. During applanation tonometry the sensor was placed on the patient’s skin over the radial artery. This placement applies pressure on the radial artery and the pressure of the sensor flattens (i.e., ‘applanates’) the radial artery. Under optimal ‘applanation conditions’, the transmural pressure of the radial artery wall is zero and the intraluminal applanation tonometry can be recorded by the sensor. In the present study applanation tonometry parameters were recorded at the left radial artery. Using applanation tonometry we registered systolic and diastolic aortic pressure, aortic pulse pressure, peripherial pulse pressure, augmentation pressure, augmentation index normalized on actual heart rate (Alx@HR) and ejection duration. All measurements were performed by the same experienced operator (DP).

Transcranial Doppler (TCD) measurements were performed using Rimed Digilite transcranial Doppler sonography (Rimed Ltd., Israel). The temporal window was used for insonation of the middle cerebral artery at 45–55 mm depth, depending on the best signal. A fixed probe was used to register systolic, diastolic and mean blood flow velocities; pulsatility indices were calculated by the device.

### Study protocol

Preoperative measurements were recorded one hour before anesthetic induction in supine position in quiet environment. Patients were then premedicated with 7.5 mg midazolam orally 60 min before entering the operating room. Anaesthesia was induced with a combination of 2 mg.kg^− 1^ propofol and 2 μg.kg^− 1^ fentanyl. Intubation was facilitated by administration of 0.6 mg.kg^− 1^ rocuronium. Fentanyl and rocuronium were repeated as needed. Anesthesia was maintained with sevoflurane in 40% oxygen. The concentration of sevoflurane was titrated to a target Bispectral Index (BIS) between 40 and 60 (Covidien, Dublin, Ireland) and it was targeted at 1 MAC. Neuromuscular block was monitored with acceleromyography (TOF Watch SX, NV Organon, Oss, the Netherlands). Standard monitoring included five-leads ECG, NIBP, core temperature at the tympanic membrane, and pulse oximetry. Normothermia was maintained with forced-air system (Bair Hugger 750, 3 M, Eden Prairie, MN, USA).

Patients were ventilated using volume-controlled square-wave flow pattern ventilation with 6–8 mL.kg^− 1^ TV, 5 cmH_2_O PEEP, 2 L.min^− 1^ fresh gas flow and with a respiratory rate adjusted to reach and maintain 40 mmHg EtCO_2_ (Draeger Primus anesthesia workstation, Draeger Lübeck, Germany).

After induction of general anaesthesia and intubation, lungs were ventilated in supine position for 20 min as described above. At this stage, hemodynamic, transcranial Doppler, applanation tonometry and ventilatory parameters were simultaneously recorded.

In the second phase of the study minute ventilation (respiratory rate and tidal volume) was changed to reach and maintain 35 mmHg EtCO_2_. After 5 min stabilisation period all measurements were repeated.

**Fig. 1:**
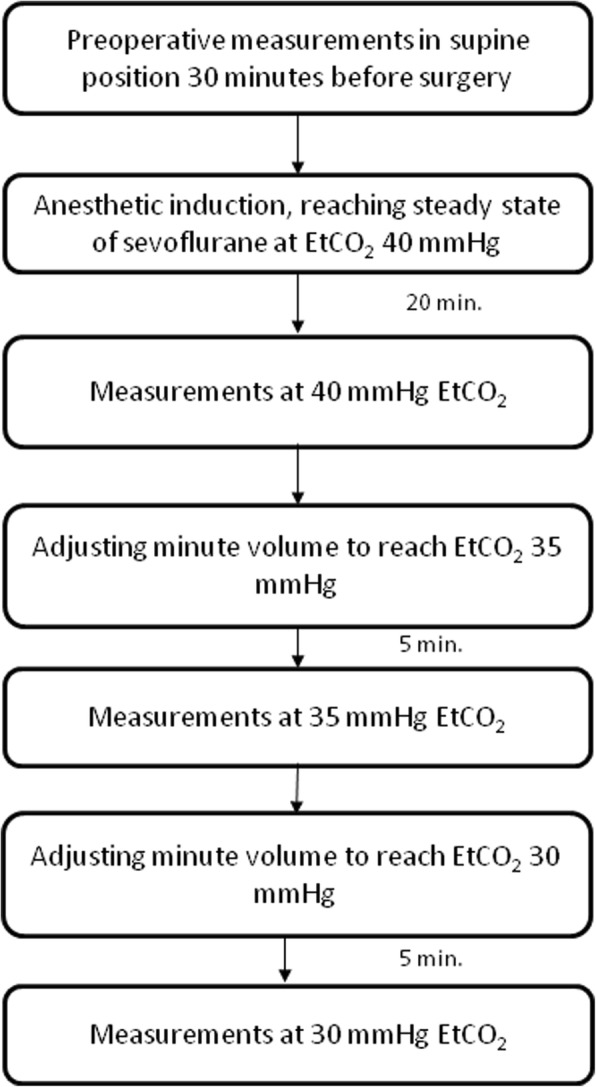
Flow chart of the study

At the next stage of measurement series ventilatory settings were again changed to reach and maintain 30 mmHg EtCO_2_ After a 5 min stabilisation period, applanation tonometry and transcranial Doppler measurements were repeated. The flow chart of the study is summarized in Fig.1.

During each measurement pulse oxygen saturation (SpO_2_), heart rate, systolic and diastolic blood pressure, bispectral index values, systolic, diastolic and mean blood flow velocities and pulsatility indices in the middle cerebral artery, systolic and diastolic aortic pressure, aortic pulse pressure, peripherial pulse pressure, augmentation pressure, Alx@HR, ejection duration and operation index were registered.

The entire study and its interpretation follows the CONSORT guidelines.

### Statistical methods

Before starting the study, a power analysis was performed to calculate the necessary number of patients to be included. We hypothesized that sevoflurane will not affect cerebral CO_2_-reactivity at 1 MAC (primary end point). Based on our previous study performed among non-anesthetized individuals, we found that hyperventilation lasting for 1 min results in a decrease of PCO_2_ by 7 mmhHg, accompanied by a 26.7 cm/s decrease of the middle cerebral artery mean blood flow velocity [7]. Accordingly, we calculated that 1 mmHg decrease of PCO_2_ results in a 3.84 cm/s change in blood flow velocity. As per protocol we intended to decrease CO_2_ by 10 mmHg in 2 steps, we calculated with a 38.4 cm/s (30 cm/s standard deviation) change, with a power of 0.9 and with an alpha of 0.01. Our power calculation indicated that 12 patients need to be included to test our hypothesis. As both registration methods may be operator-dependent, for sake of clarity we decided to include 30 patients. The normality of distribution of data was tested by Shapiro–Wilks test. Thereafter, differences were analyzed with the Repeated Measures Analysis of Variance (ANOVA) with Bonferroni post hoc correction. Values of *p* < 0.05 were accepted as statistically significant. Data are presented as mean (SD). MedCalc Statistical Software version 18.2.1. (MedCalc Software bvba, Ostend, Belgium) was used for statistical analy.

## Results

### Demographic data

Twenty-nine patients were enrolled aged 19–58 (mean 37), 17 female, 12 male. 7 patients underwent inguinal hiatal repair surgery, while 22 varicetomy. 1 patient was excluded due to inapproriate temporal window for TCD.

### Respiratory parameters and anesthetic depth during the procedure

During the course of the study ventilatory settings were changed at different stages of the study to reach and maintain different levels of EtCO_2_ (40, 35 and 30 mmHg respectively). Accordingly, there were significant differences in values of tidal volumes, respiratory rates. BIS values were stable during the entire procedure indicating adaequate depth of anesthesia (Table 1).

**Table 1:** Hemodynamic parameters during the course of the study

	ETCO_2_ 40	ETCO_2_ 35	ETCO_2_ 30	*p*
Tidal volume	516 (64)	550 (63)^‡‡‡^	570 (88)^‡‡‡^	*p* < 0.001
Respiratory rate	9 (2)^†††^	12 (13) ^‡‡‡^	15 (3)^‡‡‡, †††^	*p* < 0.001
BIS	42 (2)	42 (1)	42 (2)	*p* = 0.75

Data are presented as mean (SD) *: Significant difference compared to preoperative values ^‡^: Significant difference compared to EtCO_2_ 40 values ^†^: Significant difference compared to EtCO_2_ 35 values, ^#^: Significant difference compared to EtCO_2_ 30 values, *^, †, #, ‡^ indicates *p* < 0.05; **^, ††, ##, ‡‡^ indicates *p* < 0.01; ***^, †††, ###, ‡‡‡^ indicates *p* < 0.001

### Hemodynamic parameters

There were no significant differences neither in systolic blood pressure values, nor in heart rate during the course of the study. In contrast, there were significant differences in diastolic blood pressure: preoperative values were significantly higher than values measured in any other timepoints (Table 2).

**Table 2:** Hemodynamic parameters during the course of the study

	Preop	ETCO_2_ 40	ETCO_2_ 35	ETCO_2_ 30	*p*
Systolic blood pressure	109 (29)	106 (8)	108 (9)	108 (9)	*p* = 0.46
Diastolic blood pressure	81 (9)	65 (10)***	67 (10)***	66 (11)***	*p* < 0.001
Heart rate	67 (10)	70 (12)	67 (11)	69 (12)	*p* = 0.38

Data are presented as mean (SD), *: Significant difference compared to preoperative measured values, *** indicates *p* < 0.001

### Applanation tonometry parameters

**Fig. 2:**
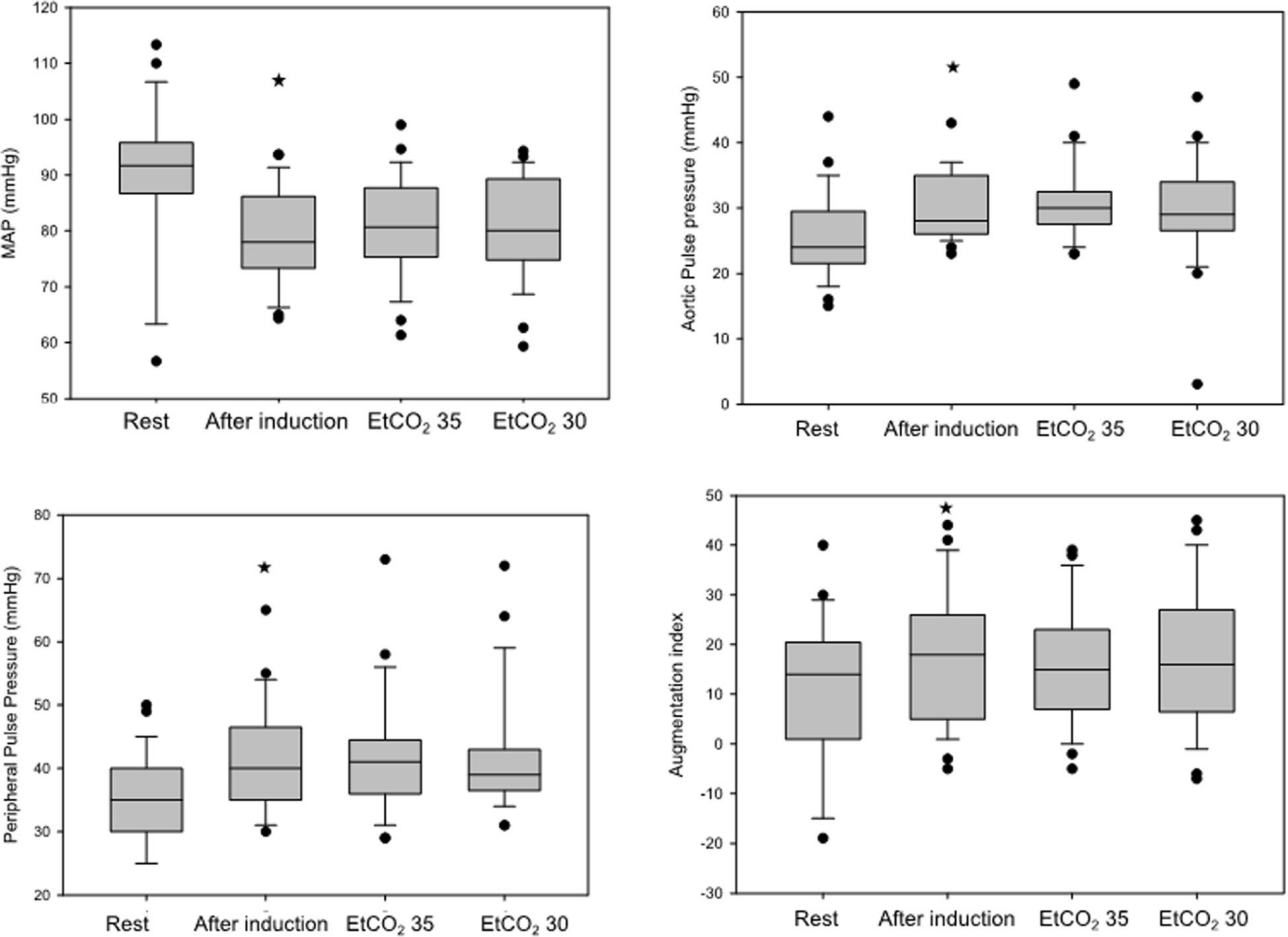
Applanation tonometry parameters at rest and during the course of the study. Medians and CI values are shown. * indicates *p* < 0.05 difference as compared to steady state values

Mean arterial pressure significantly decreased during induction of anesthesia, reaching a steady state and remained stable during the entire course of the study. In parallel, aortic and peripheral pulse pressures both increased during the induction phase as did the augmentation index. All parameters remained unchanged during the next phases of the study (Fig. 2).

### Transcranial Doppler sonography parameters

**Fig. 3:**
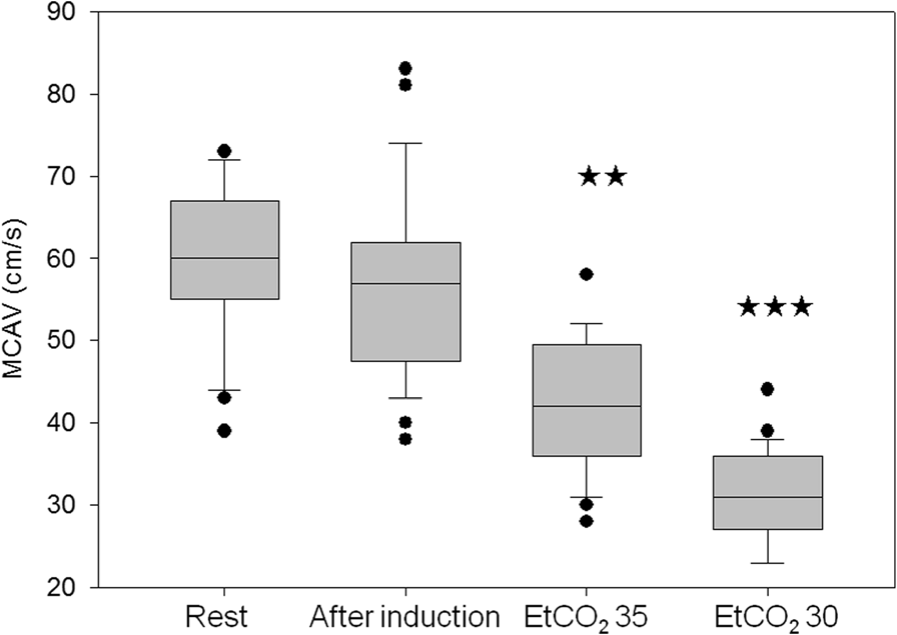
Changes of middle cerebral artery mean blood flow velocity (MCAV) during the course of the study. Medians and CI values are shown. ** indicates *p* < 0.01, *** indicates *p* < 0.001 differences as compared to steady state values

**Fig. 4:**
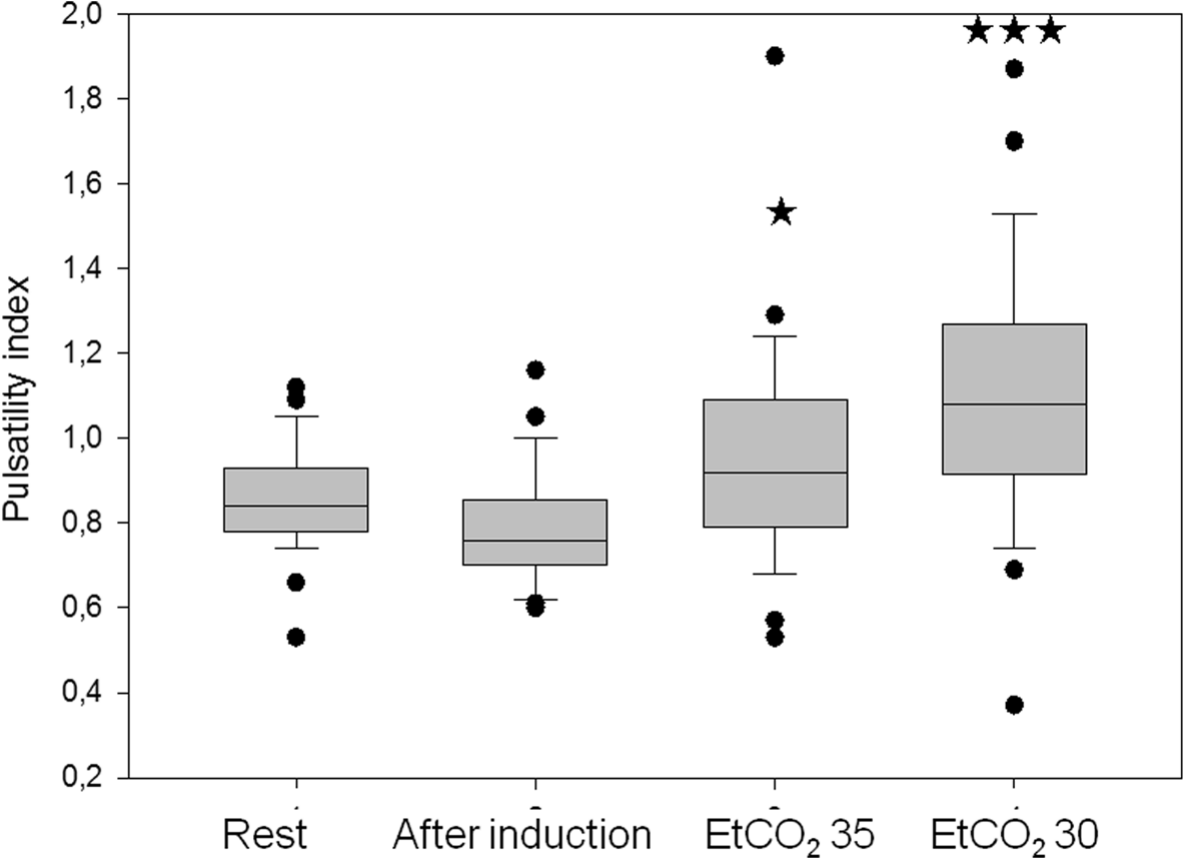
Changes of pulsatility index during the course of the study. Medians and CI values are shown. * indicates *p* < 0.05, *** indicates *p* < 0.001 differences as compared to steady state values

Preoperative middle cerebral artery mean blood flow velocity values were not significantly different from values measured at steady state sevoflurane stage under normocapnic conditions (EtCO_2_ 40 mmHg). However, mean blood flow velocity values measured during EtCO_2_ 35 and 30 mmHg were significantly different form each other as well as from preoperative and values measured at EtCO_2_40 (Fig. 3). Pulsatility index values did not change during induction phase and reaching the steady state, however they significantly increased along with the decreased EtCO_2_ values (Fig. 4.). There was a strong significant correlation between Vmean and EtCO_2_ values (*p* < 0.001, Pearson’s r = 0.79).

### Relationship between mean arterial pressure and cerebral blood flow velocity changes after induction and stabilisation of anesthesia

**Fig. 5:**
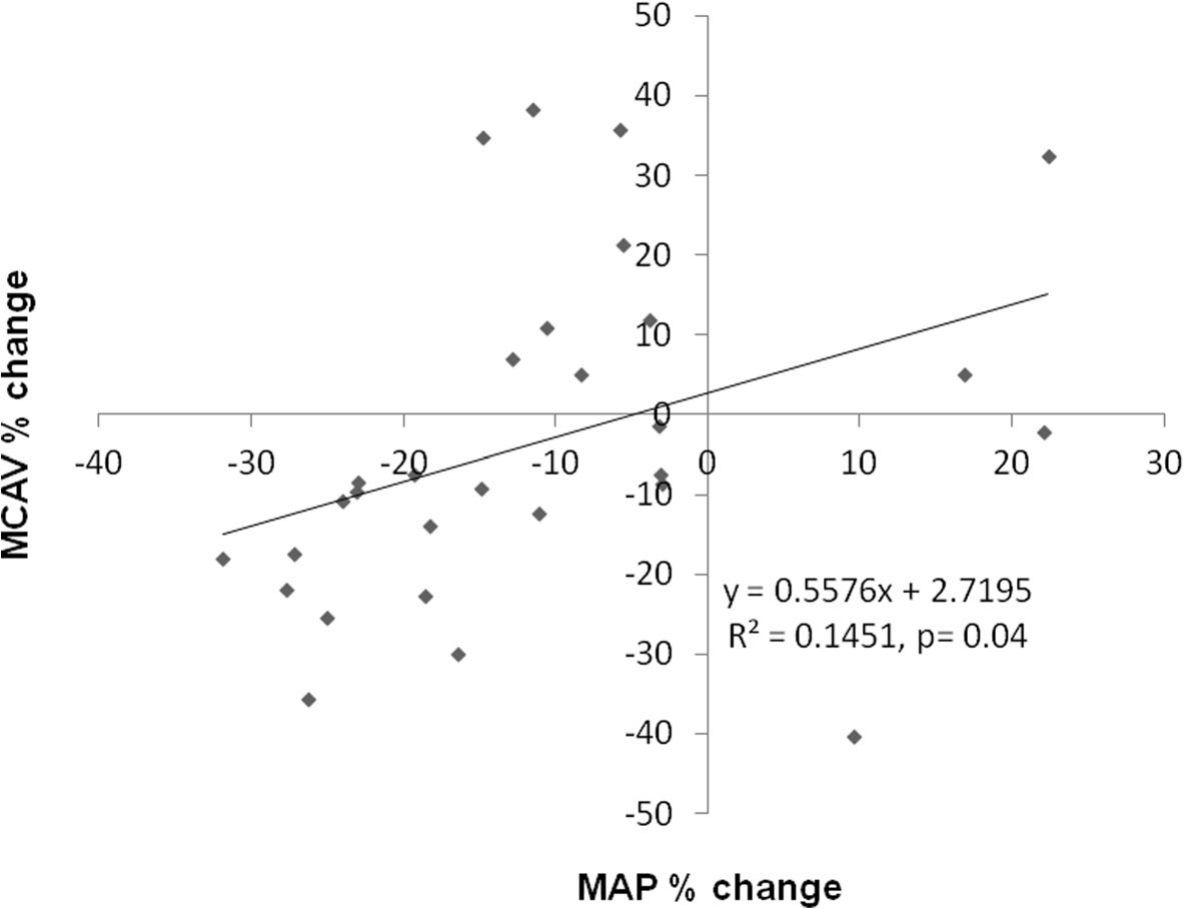
Relationship between the %change of mean arterial pressure (MAP) and middle cerebral artery mean blood flow velocity (MCAV) after anesthetic induction and reaching the steady state

We assessed the percent change between mean arterial pressure and middle cerebral artery mean blood flow velocity before anesthetic induction and 20 min after anesthesia was induced and a steady state of sevoflurane anesthesia was reached at normocapnic PCO_2_ (40 mmHg). As shown in Fig. 5, there was a significant linear relationship between the two parameters, indicating preserved static autoregulation of the cerebral circulation at normocapnic steady state of sevoflurane anesthesia.

## Discussion

**Fig. 6:**
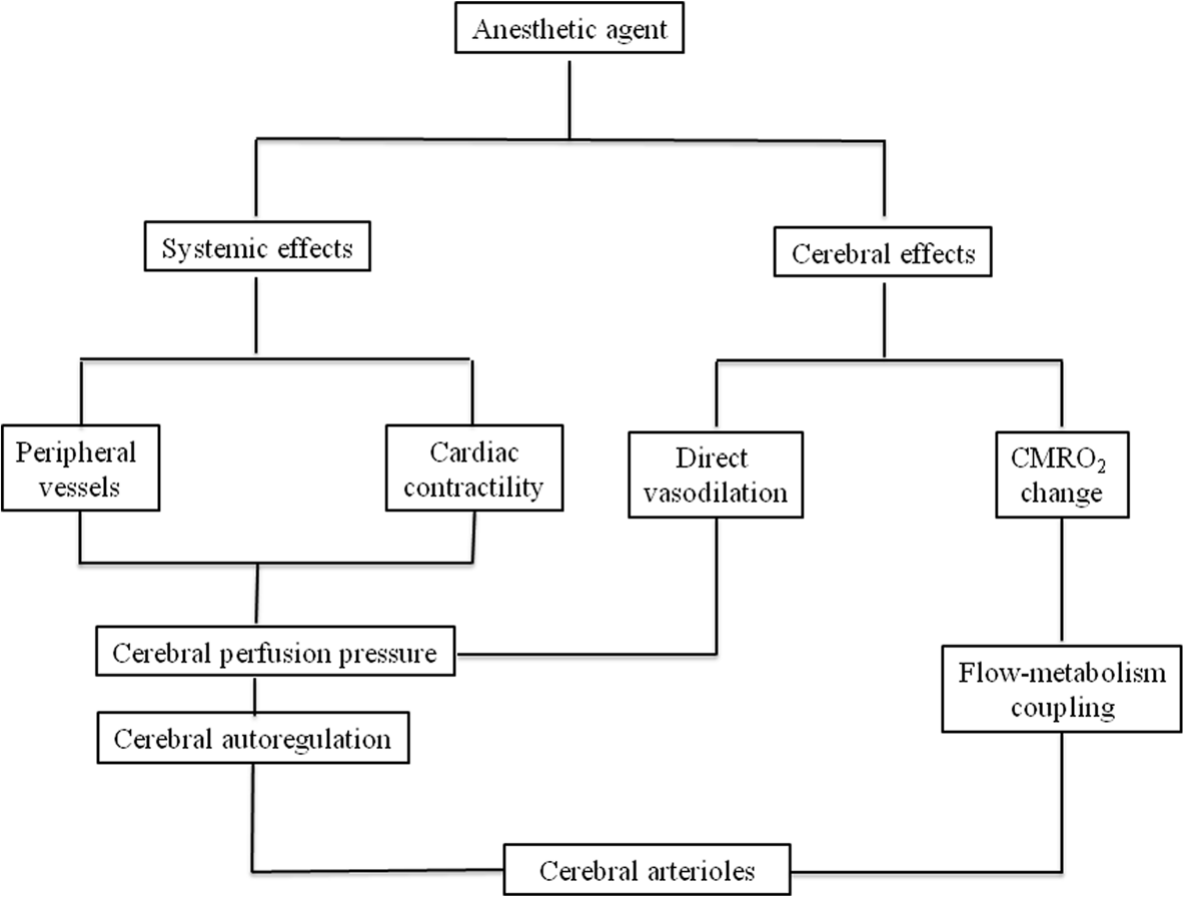
The proposed mechanism of action of anesthetics on cerebral blood flow regulation

Anesthetic agents may have various effects on systemic circulation determining the cerebral perfusion pressure. Additionally, whyle using some agents, direct cerebral effect has also to be taken into account. Both effects are dose-dependent and are exerted through vasodilation or vasoconstriction of the cerebral vessels (medium-size vessels and arterioles), as well as through decreasing CMRO_2_ - thus influencing flow-metabolism coupling of the brain tissue. The main actors of this systemic and cerebral effects are cerebral arterioles, that are responsible for autoregulatory reactions during changes of cerebral blood flow and vasoreactivity related to metabolic changes [1]. Consequently, for sake of clarity, all the possible influencing factors (systemic and metabolic factors) have to be assessed in parallel while it is intended to test the effect of an anesthetic agent on cerebral blood flow, cerebral autoregulation and cerebral vasoreactivity. The proposed mechanisms of action of general anesthetics on cerebral circulation are summarized at Fig. 6.

Previous reports indicated that sevoflurane between 0.5–1.0 MAC has minimal direct vasodilatory effect on the brain arteries [8, 9] and has no systemic hemodynamic effects (cardiac contractility and/or peripheral vascular resistance) at the most frequently used 1 MAC sevoflurane [10]. In the present study, during the induction phase, the combination of propofol-fentanyl-rocuronium and adding sevoflurane resulted in a decreased mean arterial pressure (mainly due to the decrease of the diastolic pressure) and an increase in aortic and pulse pressure. During the later phases of anesthesia, however no significant changes occurred in any of the hemodynamic parameters (mean arterial pressure, aortic pressure, pulse pressure and augmentation index), indicating no systemic effects of sevoflurane. It is conceivable that the peripheral vasodilation oserved during the induction phase can be mainly ascribed to the use of propofol.

We found that reaching the stady state under sevoflurane anesthesia resulted in a decrease of the mean arterial pressure leading to non significant change in the cerebral blood flow velocity. Cerebral autoregulation is defined as the inherent ability of the brain circulation to maintain a constant cerebral blood flow during changes of cerebral perfusion pressure [1]. In line with this, we checked the relationship between %-changes of MAP and cerebral blood flow velocity values and found a linear relationship between the two factors, indicating preserved autoregulation. While mean arterial pressure decreased, cerebral blood flow velocity remained unchanged during the induction phase and reaching the steady state. As demonstrated in Fig. 5., pulsatility index (representing the vascular resistance of the cerebral arterioles) significantly decreased during this period enabling unchanged cerebral blood flow during the decrease of MAP.

Carbon-dioxide reactivity of the cerebral circulation is defined as the change in cerebral blood flow velocity per mmHg change in PCO_2_. In previous volunteer studies it has been demonstrated that forced hyperventilation lasting for 60 s results in a 38% percent decrease of the middle cerebral artery mean flood flow velocity compared to the resting BFV [7]. The accepted normal values for CO_2_-reactivity is 2–5 cm/s/mmHg [4]. In the present study a decrease of PCO_2_ from 40 mmHg to 35 mmHg resulted in an average decrease of mean blood flow velocity of 4 cm/s/mmHg, whereas to 30 mmgHg PCO_2_ resulted in a decrease of 8 cm/s/mmHg. These values correspond to the previous observations and indicate preserved CO_2_-reactivity during sevoflurane anesthesia at MAC 1 concentrations.

Both cerebral autoregulation and cerebral vasoreactivity are multifactorially determined defending mechanisms of the brain that aim to optimize global and cerebral circulation according to the actual metabolic needs. The main actors of these two mechanisms are cerebral arterioles, roughly 200 μm in diameter [1]. The patency of autoregulatory and vasoreactivity reactions depends on systemic factors (such as cerebral perfusion pressure), local metabolic demands as well as underlying diseases of the patients. Previously it has been demonstrated that cerebral autoregulation and reactivity of the cerebral vessels to induced hypocapnia may be impaired in patients with certain underlying diseases, such as hypertension and diabetes mellitus [11–14]. As a consequence, sudden intraoperative changes of cerebral perfusion pressure and/or carbon-dioxide concentration may challenge the autoregulatory and metabolic regulatory responses and thus may result in critical brain tissue oxygenation. In line with our observations, previous studies in humans also demonstrated that sevoflurane at ≤1 MAC concentrations does not affect cerebral autoregulation [15–17]. In a very recent study Sperna Weiland and co-workers found that sevoflurane does not affect the already impaired cerebral autoregulation in diabetic patients at clinicially applied doses [18].

Finally, we have to mention the limitations of our study. The methodological limitations are that transcranial Doppler does not measure cerebral blood flow, only changes of the blood flow velocities are proportional to cerebral blood flow values. Applanation tonometry that was used for monitoring the systemic hemodynamic effect is operator-dependent procedure. Therefore all measurements were performed by the same, experienced physician (DP). And finally, in the present study we only tested CO_2_-reactivity toward hypocapnic direction. This was done intentionally, as in the usual practice normocapnia is maintained during anesthesia and in specific neuroanesthetic cases only induced hypocapnia is used as the part of the intracranial pressure decreasing strategy.

## Conclusion

Sevoflurane at clinically administered doses does not have any significant systemic hemodynamic effects and does not influence static cerebral autoregulation and cerebrovascular reactivity.

## Data Availability

Data will be available upon request from the corresponding author.

## Abbreviations

CMRO2: Cerebral metabolic rate of oxygen
CO2: Carbon dioxide
EtCO2: End-tidal CO2
MAC: Minimum alveolar concentration
MAP: Mean arterial pressure
